# An annotated and critical glossary of the terminology of inclusion in healthcare and health research

**DOI:** 10.1186/s40900-020-00186-6

**Published:** 2020-04-20

**Authors:** Shahid Islam, Neil Small

**Affiliations:** Faculty of Health Studies, University of Bradford, Richmond Road, Bradford, BD7 1DP England, UK

**Keywords:** Community engagement, Community involvement, Participation, Community development, Community empowerment, Community readiness, Co-production

## Abstract

The importance of including members of the public has been accorded a significant position in health planning, service delivery and research. But this position masks a lack of clarity about terms that are used. This paper identifies terms that are in common use in the lexicon of community based involvement and engagement in health with the intention of clarifying meaning and thus reducing ambiguity. We define and distinguish between key terms related to inclusion, we consider the terminology attached to community processes and to the challenges of inclusion and we engage with the strengths and weaknesses of the commonly used metaphor of “a ladder of participation”. We wish to contribute to the clear communication of intentions, challenges and achievements in pursuing varied forms of inclusion in health.

## Plain English summary

Interest in members of the public taking part in health research and service design has grown in recent decades. With this growth in interest there has been an increase in the number of terms used to describe how people can play an active role. For members of the public, researchers and practitioners there is a need to be able to understand what these terms mean and how one is different from another. It is not uncommon to find the same word used in different ways. For example, engagement, involvement and participation are sometimes used interchangeably. We argue that terms such as these have specific meanings. In this article we define key terms, highlight when there is a conflict in the ways different people and organisations have used them and offer a brief commentary on where such terms originate. In doing this our intention is to bring clarity to the words and ideas that appear in policies, research papers and conversations about including members of the public in health care and health research.

## Background

In recent decades we have witnessed a change in the role members of the public play in healthcare service delivery and research. At least rhetorically they have moved from being considered ‘passive recipients’, where they had minimal influence over services or research processes, towards a world where they are encouraged to be ‘active citizens’, where strap lines such as ‘nothing about us without us’ [1] and ‘big society’ [2] frequently appear in policy documents across a range of disciplines. The evidence for this change can be gauged from proxy measures including the number of papers published on patient and public involvement as they have increased ten-fold in the first 10 years of this century [3], and the number of grant funding bodies which have now mandated all applicants to demonstrate how they will include patients and members of the public in their research plans [4]. Patient and public involvement is now an embedded part of the health research eco-system, although the degree to which it is effectively pursued varies [3]. There are numerous rewards to be reaped through pursuing and implementing community inclusive approaches and these have been documented in a number of systematic reviews (see for example [5–7]. The motivations to pursue community inclusive approaches vary in their detail but a summary from Public Health England captures a commonly shared position:

Communities, both place-based and where people share a common identity or affinity, have a vital contribution to make to improve health and wellbeing. Community life, social connections, supportive relationships and having a voice in local decisions are all factors that underpin good health [8](p.4).

Given the increasingly positive zeitgeist for community inclusive approaches, and with a growing evidence base to support their impact on health and wellbeing [9], it is timely to note that participatory approaches have their origins in two very distinct agendas. The first is as an adjunct to the introduction of market principles into health policy, in the UK exemplified by “The Patients Charter” (1991) [10]. This agenda sought to promote the rights of patients as consumers – rights lodged in the belief that they should have “voice” and “choice” in relation to the health care they receive. This is an agenda that is based on an inherent individualism in making consumption choices, that is we make these choices for ourselves. The second agenda was to enhance inclusivity, in the UK the “Local Voices” initiative of 1992 is an example [11]. This agenda has been linked to a communitarian philosophy [2] which assumes – or at least hopes – that stronger, more civic-minded communities can contribute to making life better for local people whether the focus is on health, social care, the ageing population or people’s happiness and quality of life [2]. Etzioni’s [12] work, which is associated with this philosophy, similarly argued for policy choices that emphasised social solidarity in pursuit of the common good. Not surprisingly, given their different starting points, these distinct agendas do not always sit comfortably together, something Beresford [13] critically discusses, although he uses the slightly different terminology of ‘consumerist’ and ‘democratic approaches’.

Contrasting the different agendas in market based and communitarian approaches also underlines a danger in too simply conflating patient and public. These two constituencies for involvement are likely to have different priorities, for example the importance given to the provision of treatment against the allocation of resources to prevention.

However, despite these different motives and starting points, there is no doubt that public and user involvement is an approach that now occupies a position of discursive privilege; it is present in a plethora of policies [14], standards [15], guidelines [16], institutional protocols [17] and publications [9, 18] and its impact on practice has been described in a recent systematic review as *“*central to the reform of Western economies” [6] (p.2).

As the commitment to participatory approaches has grown, so too has the call for robust definitions which outline and differentiate processes that fall under this rubric. This is crucial given the World Health Organisation's (WHO) observation that, “the quantity of terms and the lack of precision with which they are employed can cause confusion” [19] (p.10). We offer a glossary of and critical commentary on salient terms found in discussions relating to inclusion of communities in health service planning, delivery and research. These terms include, amongst others, those that are ubiquitous in the discourse and literature including community development, empowerment, involvement, co-production and social exclusion.

## The terminology of inclusion

The term ‘Community’ has been used to describe the bonds, identities, relationships and interests that join people together or give them a shared stake in a place, service, culture or activity [20]. It is a term that is seen to invoke a more significant relationship than that of, “the more formal, more abstract and more instrumental relationship of state, or of society” [21] (p.76) [22]. It has been linked with terms like “interests” or “politics” in a way that reinforces its use as a “warmly persuasive word to describe an existing set of relationships”, or to encapsulate an aspired for “alternative set of relationships” [21] (p.76). According to guidance from the UK’s National Institute for Health and Care Excellence (NICE), the range and scope of the word allows communities to be defined by: “geographical location, race, ethnicity, age, occupation, a shared interest or affinity (such as religion and faith) or other common bonds, such as health need or disadvantage” [23](p.6). A research study by MacQueen et al. [24] recruited an impressively diverse sample of respondents to try and answer the question “what does community mean to you” (p1929) and found consensus forming around the following description: “a group of people with diverse characteristics who are linked by social ties, share common perspectives, and engage in joint action in geographical locations or settings” (p1929).

### Engagement, involvement and participation

There are important differences between engagement, involvement, and participation but these terms are often used interchangeably. This is noted by the WHO; “Although it is not surprising that different people understand the term community participation very differently, this diversity of understandings can cause difficulty (and) can sometimes imply that the meaning of community participation is self-evident” [19] (p.10). Marjanovic et al. [25] highlight that the distinction between key terms is particularly blurred in the fields of applied research and health services research, and as a consequence, “many systematic reviews of PPI (Patient and Public Involvement) activities note the challenges they faced finding relevant literature, in part because PPI lacks standard terminology” (p1).

Community engagement is defined as “a process of working collaboratively with groups of people who are affiliated by geographic proximity, special interests, or similar situations, with respect to issues affecting their well-being” [18](p.1). A similar definition is reached when this issue is explored by a Scientific Consortium as they describe community engagement as “the process of working collaboratively with and through groups of people affiliated by geographic proximity, special interest, or similar situations to address issues affecting the well-being of those people” [26] (p.3). There is nothing in either of these definitions which shows how much collaboration is required to warrant this being considered engagement. Moreover, it is not clear what defining characteristic sets engagement apart from involvement and participation. This perplexity was noted by INVOLVE, a UK government funded group which was established over 20 years ago to actively support input by members of the public in health and social care research. It is worth quoting how INVOLVE make efforts to resolve this problem:

“INVOLVE Advisory Group members met for a two-day symposium ( …) and one of the many discussions we had was about the confusion language can create, for example what we mean when we use the terms ‘public’ ‘involvement’, ‘engagement’ and ‘participation’ in research and how others may have a different understanding for the same words. This was also raised in our recent webinar on public involvement in social care research” [27].

These efforts produced a definition and examples on what constitutes engagement. As INVOLVE describe it, engagement is:

(W) here information and knowledge about research is provided and disseminated. Examples of engagement are:

- science festivals open to the public with debates and discussions on research
- open day at a research centre where members of the public are invited to find out about research
- raising awareness of research through media such as television programmes, newspapers and social media
- dissemination to research participants, colleagues or members of the public on the findings of a study [27].

In this way communities may be considered engaged when they are informed about a specific initiative, say, through attendance at an event or through social media without the community necessarily reciprocating or having to take an active role in the process.. Thus a defining characteristic of engagement is minimal input by the target group and no/low expectation of any reciprocity within the processes employed.

Community involvement**.** The term involvement “implies being included as a necessary part of something” [19](p.10). INVOLVE, defines involvement as being where people are actively involved in research projects and in research organisations [27]. The same criteria applies when considering involvement in service design and delivery. The essential difference between engagement and involvement, from the definition provided by INVOLVE [27], means that the former does not require members of the community to play an active role whereas with involvement it is a prerequisite.

Community participation is defined by the WHO as “a process by which people are enabled to become actively and genuinely involved in defining the issues of concern to them, in making decisions about factors that affect their lives, in formulating and implementing policies, in planning, developing and delivering services and in taking action to achieve change” [19](p.10). As such, participation requires involvement but involvement, by itself, is not enough to qualify as participation.

A different meaning is reached by INVOLVE [27] as they identify participation as being where people take part in a research study. But this use of “participation” narrows it to a scenario where members of the public are essentially subjects of research. Their influence over study design or delivery may be negligible. This ambiguity in how to define participation seems to exemplify that distinction we introduced earlier between a consumerist and a communitarian understanding, with, in this case, INVOLVE representing the former and the WHO the latter.

More generally it is clear that classification is fraught with difficulties. It is common to find research documentation (e.g. ethics application forms and grant applications) describing participation as involvement, or talking of activities in the section dedicated to Patient and Public Involvement in which people who simply participate in a study are described as being involved. The National Institute for Health Research has taken steps to ameliorate this confusion by offering the following advice in their guidance to people applying for their grants:

The term involvement refers to an active partnership between patients, members of the public and researchers in the research process. This can include, for example, involvement in the choice of research topics, assisting in the design, advising on the research project or in carrying out the research. In this section it is important that you describe in as much detail as possible how patients and the public have been involved in the development of the application as well as plans for involvement in the proposed research(...) Please note that this section does not refer to the recruitment of patients or members of the public as participants in the research [28](p.7)

### Community development and empowerment

Community development and community empowerment are sometimes conflated which is why a review by Campbell et al. [29] found a “great deal of confusion and contention in the literature”(p.67).

The inherent difficulty with defining community development was noted by Biddle in 1966 in his paper titled ‘the fuzziness of definition of community development’ [30]. The problem, as Biddle puts it, is: “(t) wo enthusiasts for Community Development, in conversation, will often discover that they are talking about quite different experiences, even though they both lay claim to the admired title” [30] (p.5). Biddle’s paper discusses the varying ways in which this term has been applied and then reaches a working definition: “Community Development is a social process by which human beings can become more competent to live with and gain some control over local aspects of a frustrating and changing world” [30](p.12).

To fully appreciate community development it is crucial to understand both its functionality and it’s ethos. Ledwith [31] notes “community development is rooted in a vision of a more fair and just world” (p.5) which is why it is considered a value based process which aims to redress imbalances in power and seeks to bring change founded on social justice, equality and inclusion [32] Operationally, community members identify issues and determine joint actions with institutions or professionals to build healthy, sustainable and equitable communities [8]. This may be achieved, for example, by influencing budgets, strategies and policies that could achieve community level improvements.

Community empowerment differs from development in that when it is in place joint actions with institutions and professionals will not be necessary as power to make such decisions will *ipso facto* already belong to the community. Community empowerment can be described as, “the ‘ultimate’ form of engagement, as it requires the ceding of power and control to communities who have traditionally been denied such privileges” [9]*(*p.46). Whilst this may be presented as desirable, it can prove burdensome and challenging for many communities. It requires a level of self-sufficiency and adequate governance that may be difficult to achieve or sustain, particularly when the empowered community does not have the support of an established institutional structure.

### Metaphors of “participation”

**Fig. 1:**
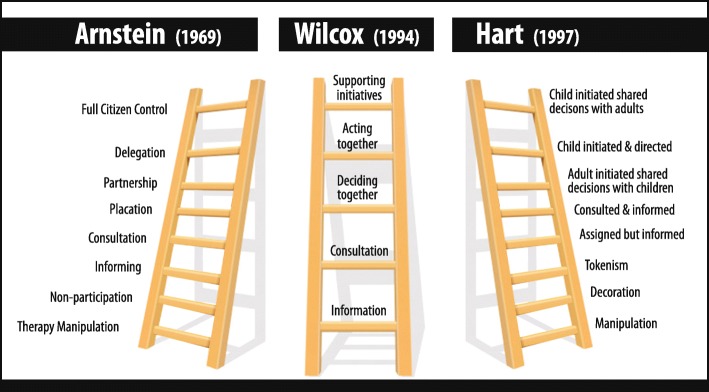
Ladders of participation

We have argued that the difference between consumerist and communitarian (or, as Beresford argues consumerist and democratic [12]) approaches to participation creates challenges for practitioners in identifying the correct nomenclature to best communicate their intentions and actions. This, in turn, creates problems for research funders. But as well as the terminology difficulties we have discussed above, there may be some confusion that stems from a metaphor that has characterised the field of inclusion. That metaphor is the ladder, the most widely recognised is Arnstein’s ladder of participation [33] which was first published in 1969. Others followed Arnsteins lead and also used the ladder metaphor. Wilcox’s ladder of participation [34] and Hart’s ladder of participation [35] are two such examples (see Fig. 1). A ladder conveniently illustrates the steps that can be taken to graduate from low levels of input through to high levels of influence and control. Activities which fit higher rungs of the various participation ladders such as ‘deciding together’, ‘shared decisions’ and ‘partnership’ may fit within the WHO definition of participation but they also demonstrate “higher” degrees of influence consistent with what we have categorised as co-production,community development and empowerment which we have shown are distinctly different to participation. The metaphor of the ladder is useful in illustrating the climb from lower to higher forms of inclusion but describing them all as “ladders of participation” risks compromising conceptual precision and, in so doing, adds to a serious communication gap in the field.

It is not only the terminology of inclusion that is complex there are also challenges in arriving at a clear and shared understanding of community processes, characteristics and challenges .

## A wider glossary of the contribution of “community” in health

### Community processes

Co-production, as a concept, emerged in the 1970’s during a study by Ostrom into the Chicago Police forces’ ambitions to improve social outcomes through working ‘with’ residents rather than ‘for’ them [36]. Ostrom was searching for a term which could encapsulate the “process through which inputs used to produce a good or service are contributed by individuals who are not ‘in’ the same organisation” [36]*(p13).* Significant hopes and aspirations are placed on this idea as one report states; “co-production looks set to create the most important revolution in public services since the Beveridge Report in 1942” [36](p.3).

A more recent definition, which has expanded the one offered above, describes co-production to mean “delivering public services in an equal and reciprocal relationship between professionals, people using services, their families and their neighbours. Where activities are co-produced in this way, both services and neighbourhoods become far more effective agents of change.” [37](p.9). Key words here are ‘equal’, ‘reciprocal’ and ‘agents of change’ which are necessary cogs of the machinery that comprise co-production; and, whilst the concept may appear straightforward to define, it has generated uncertainty and heated debate as one blogger for the Kings Fund testifies: “I regularly have to challenge very senior people and we often get stuck at some point during the process (of reaching agreement on meaning). This usually revolves around denial of one consistent and very inconvenient truth: patient involvement is not co-production” [38]. Perhaps what makes co-production particularly distinctive is the egalitarian value and philosophical underpinning it is deeply rooted in. The emphasis given to ‘equality’, ‘reciprocity’ and how people must be ‘agents of change’ means that if the above ladders were used to gauge level and depth of inclusivity then co-production would occupy a place amongst the higher rungs.

Participatory Research (PR) is an umbrella term that embraces a number of methodological approaches. A fundamental premise of PR is a recognition that marginalised groups are able to construct knowledge in meaningful ways, and that such knowledge is both valuable and significant [39]. The methodologies that fall under PR are unified by collective endeavour formulated through negotiation taking place between the researcher(s), the communities and the participants. The approaches include community based participatory research [18], participatory action research [40], cooperative inquiry [41], appreciative inquiry [42] and citizen science [43]*.* There are numerous examples of PR available in the literature. One example is where service users, health care providers and academics pursued participatory action research on a joint basis from conception of the idea through to dissemination of findings on the topic of how travel affects mental health in-patients when admitted to a hospital [44]. An important point noted in this study is the value gained through employing service users as researchers. This was considered to have achieved a two-fold benefit; one, service users as researchers were able to personally relate to and empathise with the experience they heard during interviews; and secondly, being interviewed by service users made it easier for respondents to discuss their experiences.

### Community characteristics

Community Capital. The physical and financial resources of a community are considered components of capital and there are powerful recommendations for pressing such capital into service. Community capital can be “drawn upon to generate great social value” [2](p.4) which, in turn, can energise communities into taking positive steps for health improvement. The origins of the term community capital can be traced to the theory of social capital which, according to Robert Putnam, posits that communities contain reserves that bind people together into trusted relationships through organisations and institutions where people come together to meet and socialise [20]. Building trusted relationships, from Putnam’s perspective, gives rise to a greater inclination amongst members to do things for one another. Robert Bordieu’s theory of social capital [45] takes a different position, emphasising the power of networking, building bridges with people and institutions with the purpose of gaining ‘ a credential’. Bourdieu goes on to say: “The volume of the social capital possessed by a given agent thus depends on the size of the network of connections he can effectively mobilize and on the volume of the capital (economic, cultural and symbolic) possessed in his own right by each of those to whom he is connected” (sic) [45] (p.249). Community capital incorporates both of these principles and is defined by Parsfield et al. [2] in the following way: “(W) e use it to describe the sum of Putnam’s social reserves and Bourdieu’s instrumental advantage; it is the net of social assets and resources which, if managed through the socially productive means of supporting greater social connectivity, generates benefits for the members of a community. Like any capital, it consists of a stock of valuable goods (in this case, significantly, relationships), it can be accessed by people (the members of the community in which the relationships exist) and it can be used in the production of other goods or advantages” [2] (p.21).

One practical example of community capital at work can be found in the Asset Based Community Development (ABCD) approach pioneered by Kretzmann and McKnight [42] (see Table 1).

**Table 1:** Asset Based Community Development (ABCD)

The basic tenet of ABCD is that assets exist in numerous domains in every community; these could be in individuals, associations, institutions, physical spaces, exchanges and even cultures. The ABCD approach relies on connecting these assets “to build communities from the inside out” [46]. In so doing this model reverses traditional perspectives which commonly define communities by their deficits – i.e. problems, needs, deprivation etc. The deficit approach typically begins with an outside group identifying a community’s perceived needs with a view to responding to them through programmes and interventions. This leads to stigma, it does not support any mechanisms to achieve ownership amongst community members and it denies the community the right to develop solutions from within [47].	

Community Readiness **“**is the degree to which a community is prepared to take action on an issue” [48] (p.3). Launching any new programme requires alignment between implementation efforts and the level of readiness in a community to take part in the planned programme if it is to be successful. When these variables work in harmony the possibility of acceptance and uptake by the target community increases [49]. The community readiness model (CRM), developed by researchers from the Tri-ethnic Centre in Colorado USA, was originally designed to test acceptance of a drug and alcohol prevention programme and has since developed into a toolkit [48] which has been applied to a broad range of topics across the globe including obesity in the UK [50], domestic violence in South Korea [51] and HIV prevention in Bangladesh [52]. In the original paper describing CRM, the authors highlight their concern about improper use of the terminology when they say: “Community readiness is a term that is being used more and more by various authors and in many contexts. Although different terms and descriptions can be used to describe the stages of readiness and the dimensions of readiness, it should be noted that the specific terms and descriptions have been thoroughly tested. If other names or descriptors are used, it should be incumbent on those using such terms to provide data showing that they have been subjected to an equivalent process” [49] (p.298).

Community Wellbeing**.** If communities possess anthropomorphic characteristics such as owning capital and showing a state of readiness to address issues then, in much the same way, it might be possible to identify a community’s level of wellbeing. There are a wide variety of definitions in use to describe community wellbeing. South et al. [53] state that, “community wellbeing is a complex concept, with no agreed definition(s) and many related concepts”(p.4). For example in one paper community wellbeing is described as being “about strong networks of relationships and support between people in a community” [53] (p.4) whereas another study defines community wellbeing “as the satisfaction with the local place of residence taking into account the attachment to it, the social and physical environment, and the services and facilities” [54] (p734). Despite these differences, the common unifying point is the importance of measuring determinants of health and social wellbeing at a community level. There are now several toolkits that do this (see South et al) [53].

### Challenges

Social Exclusion. The terms poverty and social exclusion are often found together as there are some important overlaps between them. Most commentaries describe relative poverty as a lack of resources which then manifest in, “the absence or inadequacy of those diets, amenities, standards, services and activities which are common or customary in society” [55] (p649). Social exclusion*,* however, is a broader concept encompassing not only low material means but the inability to participate effectively in economic, social, political and cultural life which then leads to alienation and distancing from mainstream society [56]*.* Social exclusion can lead to the exacerbation of health inequalities, for example if groups who are privileged and have easy access to services make use of what is available, whilst those who are marginalised and disenfranchised remain absent. Equally, research samples which do not adequately consider and redress social exclusion run the risk of recruiting an unrepresentative and/or biased sample.

Hidden Populations is a term which appears in the context of recruitment to programmes or sampling for research purposes. It refers to disadvantaged and disenfranchised groups that are difficult for researchers or service providers to access in a cost-efficient way, in large enough numbers, to meet the needs of the study or the service provision [57, 58]. There are many reasons for populations becoming *hidden* for example some groups of people such as drug users, illegal migrants, sex workers or ex-offenders may not wish to make themselves known to services; while other groups may have no intention to hide but find the same outcome for different reasons. For example, older people with minimal social contact, homeless people or young people excluded from school find that they are hidden from the view of providers for reasons related to the more general phenomenon of social exclusion.

Hard to Reach refers to community groups that are difficult to involve, engage or achieve adequate levels of participation in research or health delivery programmes. This is a contentious term as populations are described in ways that could suggest apathy and lead to stigma, especially since it is usually applied to describe those communities that are ‘under-served’, characteristically minority groups, those ‘slipping through the net’, and the ‘service resistant’ [59]. It is a term that “implies the problem as one within the group itself, instead of those trying to reach them” [58] (p.1). Critics have argued that no-one is hard to reach and the reality is more imaginative methods and more resources may be necessary in reaching groups who do not avail themselves of services offered. A one-size-fits-all approach to engagement which does not take account of impediments arising from social exclusion is both the cause and consequence of groups becoming hard to reach.

## Concluding remarks

Terms appearing in this paper were selected on the basis of their frequent appearance in contemporary debates and policy guidance when describing initiatives which involve working with communities to improve health and wellbeing or to research these areas. When planning this paper we were faced with a lengthy list of concepts to choose from and decided to narrow this down to those terms which we have experienced to be ambiguous and those that risk meaning different things to different people within the lexicon of inclusive approaches. Collectively the terms underline the contradictions and complexity of this area. We began by suggesting two agendas, enhancing individuals capacity as consumers of health and pursuing a common good through social solidarity. The former leaves the individual locked into a consumerist ideology. It represents the incorporation of the individual into a prevailing discourse in a way that supports this discourse rather than enhancing their agency. The latter also engages the individual and the community in a prevailing form of governmentality that can appear to be in their interests but can be effectively split off from the mechanisms by which key resource decisions are made. Governmentality here is understood as the way governments try to produce those citizens best suited to fulfil their policies [60]. The different way the term “participation” has been used illustrates the two agendas and the dangers that an intention to pursue a structural change can shift from a communitarian into an individualist approach.

Researchers and health providers need to keep a critical vigilance about the terms we use. This is important even when the terms relate to superficially desirable “inclusion” and “community” activities. Key to maintaining this vigilance is a critical engagement with language. There is a danger in conflating terms that have a distinct difference. The erosion of this difference adversely impacts on the clear communication of intentions and of challenges and achievements in pursuing the varied forms of inclusion.

## Data Availability

Data sharing is not applicable to this article as no datasets were generated or analysed during the current study.

## Abbreviations

ABCD: Asset Based Community Development
CRM: Community Readiness Model
NICE: National Institute for Health and Care Excellence
PR: Participatory Research
PPI: Patient and public involvement
WHO: World Health Organisation
